# Pooled Antibiotic Susceptibility Testing Performs Within CLSI Standards for Validation When Measured Against Broth Microdilution and Disk Diffusion Antibiotic Susceptibility Testing of Cultured Isolates

**DOI:** 10.3390/antibiotics13121214

**Published:** 2024-12-14

**Authors:** Emery Haley, Frank R. Cockerill, Rick L. Pesano, Richard A. Festa, Natalie Luke, Mohit Mathur, Xiaofei Chen, Jim Havrilla, David Baunoch

**Affiliations:** 1 Department of Clinical Research, Pathnostics, Irvine, CA 92618, USA; ehaley@pathnostics.com (E.H.); nluke@pathnostics.com (N.L.); 2 Independent Researcher, Trusted Health Advisors, Orange, CA 92675, USA; frankcockerill@trustedhealthadvisors.us (F.R.C.); rickpesano@trustedhealthadvisors.us (R.L.P.); 3 Department of Research and Development, Pathnostics, Irvine, CA 92618, USA; rfesta@pathnostics.com; 4 Department of Medical Affairs, Pathnostics, Irvine, CA 92618, USA; mmathur@pathnostics.com; 5 Department of Informatics, Pathnostics, Irvine, CA 92618, USA; xchen@pathnostics.com (X.C.); jhavrilla@pathnostics.com (J.H.)

**Keywords:** urinary tract infection, pooled antibiotic susceptibility testing, antibiotic resistance, heteroresistance, disk diffusion, broth microdilution

## Abstract

**Background/Objectives**: While new methods for measuring antimicrobial susceptibility have been associated with improved patient outcomes, they should also be validated using standard protocols for error rates and other test metrics. The objective of this study was to validate a novel susceptibility assay for complicated and recurrent urinary tract infections (UTIs): pooled antibiotic susceptibility testing (P-AST). This assay was compared to broth microdilution (BMD) and disk diffusion (DD), following Clinical and Laboratory Standards Institute (CLSI) guidelines for assessment of error rates and agreement. **Methods**: This study analyzed consecutive fresh clinical urine specimens submitted for UTI diagnostic testing. Upon receipt, the urine samples were subjected in parallel to standard urine culture and multiplex polymerase chain reaction (M-PCR) for microbial identification and quantification. Specimens with the same monomicrobial non-fastidious bacteria detected by both M-PCR and standard urine culture (SUC) underwent standard antibiotic susceptibility testing (AST) and P-AST antibiotic susceptibility testing. Analysis was also undertaken to assess the presence of heteroresistance for specimens with P-AST-resistant and BMD/DD consensus-susceptible results. **Results**: The performance measures without correction for heteroresistance showed essential agreement (EA%) of ≥90%, very major errors (VMEs) of <1.5%, and major errors (MEs) of <3.0% for P-AST, all meeting the threshold guidelines established by CLSI for AST. The categorical agreement (CA%) also met acceptable criteria (>88%), as the majority of the errors were minor (mEs) with essential agreement. The very major and major error rates for P-AST decreased to <1.0% when heteroresistance was accounted for. **Conclusions:** The P-AST assay methodology is validated within acceptable parameters when compared to broth microdilution and disk diffusion using CLSI criteria.

## 1. Introduction

With antimicrobial resistance on the rise, including 2.8 million antimicrobial-resistant infections reported annually in the US, antibiotic susceptibility testing (AST) is an essential part of the work of clinical microbiology laboratories, with the results increasingly informing clinicians prescribing decisions for infectious diseases [1]. In particular, antimicrobial-resistant organisms causing urinary tract infections (UTIs) have become an increasingly dire problem, contributing to an estimated one-quarter million deaths globally in 2019 [2]. Based on the routine clinical use of standard urine culture (SUC), UTIs have been presumed to be primarily caused by *E. coli* and a few other classically recognized Gram-negative uropathogens, though newer research with molecular-based microorganism detection has shown that several emerging and/or opportunistic uropathogens, including fastidious, anaerobic, slow-growing, and Gram-positive organisms, are also common in UTIs [3,4,5,6,7,8].

AST methods are continually evolving and now include matrix-assisted laser desorption/ionization time-of-flight (MALDI-TOF) mass spectrometry, novel microfluidics, polymerase chain reaction (PCR), gene sequencing, and more [9]. With a high discordance between resistance gene results and actual resistance phenotypes [10,11], phenotypic AST results are a critical component for accurate management. Many clinical microbiology labs utilize automated systems, such as VITEK, Phoenix, and MicroScan systems, for these assays but the results can vary widely according to the software versions and cards used [12]. As new methods for measuring AST are developed, they need to be validated against standard techniques to demonstrate low error rates.

### 1.1. Current Standard AST Technologies

Due to its low cost and relative ease of use, the disk diffusion (DD) method is the most widely used phenotypic AST method in microbiology laboratories globally, with clinical breakpoint standards available for the most common and clinically relevant bacterial pathogens [13]. It is one standard method used for comparison in validating new technologies. However, DD AST is a qualitative technique and is unable to determine quantitative minimum inhibitory concentration (MIC) values.

The preferential standard against which new methods and systems are typically validated is the broth microdilution (BMD) method [14]. The BMD method is performed in 96-well microtiter plates, allowing several antimicrobial substances and dilutions to be conveniently tested in one plate [13]. The BMD AST is typically performed using inoculates from one to three isolates (colonies) for each species of pathogen detected [15]. Since all the methods have significant variability, a common method of validation is to confirm the results of BMD with DD when called for.

### 1.2. Pooled Antibiotic Susceptibility Testing (P-AST)

A more recently developed technique, P-AST, is a phenotypic microtiter plate-based assay that measures microbial growth in the presence of antibiotics at multiple dilutions using resazurin as a fluorescent probe. It is a component of the M-PCR/P-AST assay (Guidance^®^ UTI, Pathnostics, Irvine, CA, USA), performed on all samples in which a non-fastidious bacterium is identified (see Supplemental Table S1 for details of targeted organisms and list of fastidious organisms). Rather than having the sample plated on solid media, like standard culture, urine samples are grown in Mueller–Hinton broth (MH) and distributed into the wells of a plate that contain different dilutions of UTI-relevant antibiotics [16]. Antibiotic MIC and categorical interpretations (resistant, sensitive, or intermediate) are reported consistently with Clinical and Laboratory Standards Institute (CLSI) guidelines. This novel method seeks to address the limitations of AST, with prior research showing significantly improved patient outcomes when this diagnostic test was used for complicated and recurrent UTIs [17,18]. These publications have reported a significant reduction in hospitalizations, emergency room (ER) and urgent care visits, and UTI recurrence, along with a reduction in empiric therapy prescriptions when the M-PCR/P-AST assay was utilized [17,18]. This assay has also shown a quicker turnaround time [17] and improved detection of urine pathogens in symptomatic UTI cases. Multiplex polymerase chain reaction (M-PCR)-positive specimens with negative or “mixed flora”/“contaminated” SUC results have demonstrated a significant positive association with urine inflammatory biomarkers [8,19], indicating the high specificity of the M-PCR/P-AST assay in addition to the higher sensitivity of the assay compared to urine culture [20,21,22].

### 1.3. Diagnostic Gap for Complicated and Recurrent UTI

The current standard of care utilizing SUC as the diagnostic for recurrent and complicated UTIs is leading to hundreds of thousands of hospitalizations, with over 25% of sepsis cases being due to urosepsis [23]. UTI management also has a high rate of empiric therapy use in the majority of patients, likely due to long turn-around times for SUC results and the significant number of negative or mixed flora/contamination results [17].

The current AST methods and standard urine culture with AST (SUC), which has been the standard of care UTI diagnostic for decades, have limitations, including the inability to account for antimicrobial resistance in cases without organism growth, polymicrobial infections with mixed flora/contamination results, and cases with heteroresistance [20,21,22]. Poor outcomes have been associated with negative standard urine culture results in UTI symptomatic cases, and those cases showed pathogen growth when plated on expanded culture plates [18,24]. The limitations of SUC to identify more than two species is a significant issue for polymicrobial UTI infections, which make up a significant fraction of UTIs, according to both expanded culture and molecular-based microorganism detection methods employed in both voided and catheter-collected urine specimens from symptomatic individuals [7,8,17,24,25].

Heteroresistance is a type of heterogeneity in which drastically different antimicrobial susceptibilities exist within a single microbial clone, termed “monomicrobial heteroresistance” [26]. Such a clone must contain two or more genetically “identical” [27] yet phenotypically distinct sub-populations [28]. The heteroresistance phenotype is attributed to unstable or transient variations in gene expression, which can result from epigenetic regulation [29,30], epigenetic inheritance [31], or transient genetic mutations [32], such as point mutations in regulatory genes [33] or increased copy numbers of antibiotic resistance genes [15]. The failure to efficiently detect and analyze heteroresistance has been posited to be both a driver of classical homogeneous resistance [34] and a cause of unexplained clinical antibiotic treatment failures [35,36].

### 1.4. Objectives

In this study, we aimed to validate P-AST in monomicrobial cases using the standards established by CLSI. We measured error rates and agreement when comparing the assay in parallel to standard methods of BMD and DD, using urine samples obtained from patients with a UTI. We analyzed cases where both SUC and PCR identified a single urine pathogen to assess if the P-AST method of measuring microbial growth in MH broth using fluorescence was valid. These cases were analyzed both with and without correcting for heteroresistance.

## 2. Results

This study included monomicrobial urine specimens from 250 subjects. The subjects ranged in age from 28.0 to 100.5 years (median = 74.9) with an average age of 74.6 years (SD = 10.2). The subjects were 54.8% female (*n* = 137) and 45.2% male (*n* = 113). Each specimen was submitted with at least one ICD-10-CM code associated with a suspected UTI diagnosis, and some specimens were submitted with multiple relevant ICD-10-CM codes (38 had two ICD-10-CM codes, eight had three ICD-10-CM codes, one had four ICD-10-CM codes, and five had five ICD-10-CM codes). The most common of these codes are outlined in Table 1.

### 2.1. Analysis Without Correction for Heteroresistance

#### 2.1.1. All Organisms

A total of 250 specimens with monomicrobial non-fastidious organisms were included (Table 2).

Each specimen was tested against up to 19 antibiotics (organism-dependent), resulting in 4594 organism–antibiotic combinations for comparison of both antibiotic susceptibility test method results (Table 3). The metrics for P-AST following CLSI criteria are shown in Table 4. Essential agreement, VMEs, and MEs are within CLSI criteria. Minor errors are allowed by CLSI if they fall within lab set criteria, especially when most are minor errors with essential agreement. The categorical agreement is close to 90%, with disagreements largely due to minor errors with essential agreement, and is also acceptable under CLSI.

#### 2.1.2. All Monomicrobial *E. coli* Cases

A total of 126 specimens with monomicrobial *E. coli*, each tested against 19 antibiotics, resulted in 2326 organism–antibiotic combinations for comparison of both antibiotic susceptibility test method results (Table 5). The metrics for P-AST are shown in Table 6, showing acceptable error rates and agreement.

#### 2.1.3. All Non-*E. coli* Monomicrobial Organism Cases

A total of 124 specimens with monomicrobial organisms other than *E. coli*, each tested against up to 19 antibiotics (organism-dependent), resulted in 2268 organism–antibiotic combinations for comparison of both antibiotic susceptibility test method results (Table 7). The metrics for P-AST are shown in Table 8, showing acceptable error rates and agreement.

#### 2.1.4. All Gram-Negative Monomicrobial Organism Cases

A total of 200 specimens with a monomicrobial Gram-negative bacteria species, each tested against up to 19 antibiotics (organism-dependent), resulted in 3677 organism–antibiotic combinations for comparison of both antibiotic susceptibility test method results (Table 9). The metrics for P-AST are shown in Table 10, showing acceptable error rates and agreement.

#### 2.1.5. All Gram-Negative Monomicrobial Organism Cases, Excluding *E. coli*

A total of 74 specimens with a monomicrobial Gram-negative bacterial species other than *E. coli*, each tested against up to 19 antibiotics (organism-dependent), resulted in 1351 organism–antibiotic combinations for comparison of both antibiotic susceptibility test method results (Table 11). The metrics for P-AST are shown in Table 12, showing acceptable error rates and agreement.

#### 2.1.6. All Gram-Positive Monomicrobial Organism Cases

A total of 50 specimens with a monomicrobial Gram-positive bacterial species, each tested against up to 19 antibiotics (organism-dependent), resulted in 917 organism–antibiotic combinations for comparison of both antibiotic susceptibility test method results (Table 13). The metrics for P-AST are shown in Table 14, showing acceptable error rates and agreement.

#### 2.1.7. Analysis with Correction for Heteroresistance

For all 250 monomicrobial specimens, 65 instances of heteroresistance were detected using the workflow detailed in the Methods section. Table 15a is the contingency table for the P-AST results, and Table 15b is the contingency table for BMD AST, based on correction for heteroresistance. Very major errors and major errors by P-AST decreased to <1.0% after correcting for the 65 instances of heteroresistance. These cases were categorized as BMD isolate AST VMEs (Table 16).

In all, heteroresistance was demonstrated in 38 organism–antibiotic combinations. *E. coli* demonstrated heteroresistance to the most antibiotics, while Enterobacter group organisms (which includes *K. aerogenes* and *E. cloacae*) exhibited the highest relative rate of heteroresistance per antibiotic (Table 17).

## 3. Discussion

Clinicians are increasingly relying on AST to inform their prescribing decisions for drug-resistant infectious diseases, including UTI [1]. The current standard of care using SUC has led to poor outcomes for complicated and recurrent UTIs, including hospitalization and sepsis.

Traditional isolate AST methods have been used as the clinical standard for many types of infectious disease to guide treatment decisions for decades. However, several limitations of these techniques have also been recognized, which become more significant in complicated/recurrent UTIs and polymicrobial cases. Automated systems commonly used by clinical microbiology labs, such as VITEK, Phoenix, and MicroScan systems, exhibit high result variability due to the variety of software versions and cards used [12]. Disk diffusion (DD) AST, the most common method used globally [13], is unable to determine quantitative MIC values [14]. Both BMD and DD isolate AST methods frequently require a turnaround time of multiple days [17]. Urine culture often fails to detect resistant subpopulations at low frequencies, which are the samples with heteroresistance [37]. This inability to efficiently detect and analyze heteroresistant bacterial populations has been posited to be both a driver of classical homogeneous resistance [34] and a cause of unexplained clinical antibiotic treatment failures [35,36]. Finally, isolate AST methods cannot account for antibiotic susceptibility arising from multispecies interactions in polymicrobial infections [38,39,40,41,42,43,44,45,46,47]. Polymicrobial infections have been reported in up to 52% of suspected urinary tract infection (UTI) cases in older adult populations [5,48,49,50] and have specifically been associated with poorer outcomes [51]. These limitations create a significant clinical gap, particularly for patients with recurrent and complicated UTIs. This population needs an alternative that can improve patient outcomes and reduce empiric therapy rates.

P-AST is a novel AST method that aims to fill many of these gaps. It does provide quantitative MIC values and a rapid turnaround time [17] and is designed to account for both heteroresistance and for effects of multi-species interactions that may alter susceptibility in polymicrobial infections. There is prior evidence showing improvements in patient outcomes associated with Guidance UTI, which is the M-PCR/P-AST assay that P-AST is a component of [17,18]. However, it is important to generate validation data on P-AST due to its novel nature compared to the long-used standard practice of isolate testing.

In this study, we aimed to validate P-AST by comparing its results to isolate AST from the same clinical urine specimens. This analysis was focused on monomicrobial samples where SUC was able to identify the organism, since these cases were likely to have SUC AST results and P-AST match, when heteroresistance is not present, if the P-AST assay is a valid method to use.

Using CLSI standard validation protocols, we found that the rates of very major errors were <1.5% and major errors were <3.0% for the P-AST method prior to correcting for heteroresistance, meeting the threshold in the CLSI guidelines for AST verification and validation studies [36]. Minor errors were also low (9.0% overall), in line with our laboratory performance target threshold of ≤10%. The vast majority of these minor errors were minor errors with essential agreement (77% of minor errors in all cases). For monomicrobial *E. coli* infections, the CA% met the target performance threshold of ≥90%. Although the CA% for specimens with monomicrobial infections with organisms other than *E. coli* fell slightly below the ≥90% threshold (86.1% (CI = 84.6%, 87.5%)), this CA% was still acceptable per CLSI standards since the majority (77% (243)) of the 315 errors were minor (mEs), and 75% (182) of those minor errors had essential agreement [52,53]. Furthermore, the EA%, which compared MICs between P-AST and the “gold-standard” BMD reference method, was very high (>93%). The United States Food and Drug Administration (FDA) “Class II Special Controls Guidance Document: Antimicrobial Susceptibility Test (AST) Systems” also sets performance standards, which generally match the CLSI guidelines [54]. The one exception is that the FDA guidance document defines the statistical criteria for acceptable very major discrepancies (vmj) as “an upper 95% confidence limit for the true vmj rate of ≤7.5% and the lower 95% confidence limit for the true vmj ≤1.5%”, offering slightly more leniency for the presence of VMEs. Therefore, the P-AST assay met performance standards set forth both by the FDA and by the more stringent CLSI criteria [52,53]. 

This study was designed to strongly favor SUC by not including SUC negative and mixed flora/contamination cases, by assuming that the AST result was correct when both standard methods agreed, and by excluding cases where the standard methods disagreed. Results without correcting for heteroresistance were provided, and P-AST was within acceptable limits using CLSI standards. When heteroresistance was accounted for, the assay had fewer errors, since it was demonstrated in those cases that the standard AST incorrectly missed antibiotic resistance caused by heteroresistance [17].

This study establishes the validity, low rate of error, and accuracy of P-AST, which uses a microbial pellet and fluorescence for susceptibility testing, as measured against CLSI protocols for monomicrobial samples. This study was limited to these types of cases, and future studies will need to evaluate the performance of P-AST in polymicrobial specimens with multiple non-fastidious organisms and the impact of fastidious organisms.

## 4. Materials and Methods

### 4.1. Study Design and Identifying Candidate Specimens

This study is an analysis of consecutive fresh clinical urine specimens collected in the US and submitted with sufficient volume (minimum 2 mL) in a boric acid stabilizer for diagnostic testing, along with ICD-10-CM codes consistent with a diagnosis of UTI. Specimens with the same monomicrobial non-fastidious bacteria identified by both M-PCR and SUC at a density of ≥10,000 cells/mL or CFUs/mL were selected for analysis. Samples for the study were collected via a biobank in which remnant urine specimens remaining after routine clinical testing were de-identified and assigned a unique repository code label associated only with the subject’s age, sex, and any associated ICD-10-CM code(s). The Western Institutional Review Board deemed the use of the data to be exempt under 45 CFR § 46.104(d)(4), as the information was used in a manner in which the identity of the subject could not be readily ascertained directly or through identifiers linked to the subjects, the subject was not contacted, and the investigator did not re-identify the subjects. Consecutively received, fresh, never-frozen biobanked specimens received within the window of stability were utilized for this validation study.

### 4.2. Bacterial Identification with Multiplex-Polymerase Chain Reaction (M-PCR) (Guidance^®^ UTI, Offered by Pathnostics, Irvine, CA, USA)

The M-PCR assay was performed as previously described [17]. Briefly, DNA extracted from the urine samples using a King Fisher/MagMAX™ automated DNA extraction instrument and a MagMAX™ DNA Multi-Sample Ultra Kit (Thermo Fisher, Carlsbad, CA, USA) was mixed with a universal PCR master mix and amplified using TaqMan technology in a Life Technologies 12K Flex 112-format Open-Array System (Thermo Fisher Scientific, Wilmington, NC, USA). Probes and primers were used to detect 23 bacteria, four yeast, three bacterial groups, and 32 antibiotic resistance genes. However, only specimens with a single non-fastidious bacterial species or group were included in the current study.

### 4.3. Bacterial Identification with Standard Urine Culture (SUC)

Bacterial identification by SUC was performed as previously described [55]. Briefly, a 1 µL sterile plastic loop was used to inoculate both a blood agar plate (BAP) and a colistin and nalidixic acid agar/MacConkey agar (CNA/MAC) plate (Hardy Diagnostics, Santa Maria, CA, USA) with one loop of urine specimen on each side of the CNA/MAC plate. The plates were all incubated at 35 °C in a non-CO_2_ incubator for 16 h and examined for growth.

### 4.4. Pooled Antibiotic Susceptibility Testing (P-AST) (Guidance^®^ UTI, Offered by Pathnostics, Irvine, CA, USA)

The fluorescence-based P-AST test component determines susceptibility to 19 antibiotics commonly used for UTI treatment. The assay was performed as described previously [24]. Briefly, 1 mL of urine specimen was aliquoted into a 1.7 mL microcentrifuge tube. After centrifugation, the supernatant was aspirated and discarded, and the pellet was suspended with 1 mL of Mueller–Hinton growth (MHG) media for incubation at 35 °C in a non-CO_2_ incubator for 6 h. Samples reaching a predetermined density threshold at the end of the incubation were diluted by aliquoting an appropriate volume of the sample into a 50 mL conical tube containing 29 mL to achieve a final concentration of around 500,000 cells/mL in MHG media. Then, the diluted sample was inoculated into a 96-well plate pre-loaded with antibiotics and incubated along with the control plates for 12–16 h at 35 °C. Resazurin was used as a fluorescent probe to measure cell growth. The fluorescent density of the samples was measured on an Infinite M Nano+ Microplate Reader (TECAN, Nänikon, Switzerland).

### 4.5. Broth Microdilution (BMD) Antibiotic Susceptibility Testing

BMD AST was performed on isolates from the SUC plates following standard procedures outlined in the CLSI M07 12th edition (2024) [56]. Briefly, individual isolates were suspended in MHG media and adjusted to 0.5 McFarland. This preparation was diluted to a final density of 5 × 10^5^ CFUs/mL in MHG that was supplemented with relevant antibiotic dilutions in a 96-well microtiter plate. These plates were incubated at 35 °C in a non-CO_2_ incubator for 16 h, after which the turbidity of each well was measured against positive and negative growth controls.

### 4.6. Disk Diffusion (DD) Antibiotic Susceptibility Testing

Initial DD AST was performed on organisms isolated by SUC following the standard procedures outlined in the CLSI M100 34th edition [16]. DD AST was performed during heteroresistance analysis on a diluted subculture from a P-AST well displaying resistance. Briefly, the content of each P-AST-resistant antibiotic well was diluted (100 µL of well culture + 900 µL of fresh MH) and precultured individually overnight at 35 °C.

In every case, the suspended isolate or pre-cultured well dilution was grown as a lawn on an MH plate with a single antibiotic disk. After incubation at 35 °C for 16 h, clearance zones were measured and interpreted according to the CLSI M100 34th edition [16].

### 4.7. Four Times Antibiotic Concentration Culture for Resistance Verification

Briefly, the content of each P-AST-resistant antibiotic well was diluted (100 µL of well culture + 900 µL of fresh MH) and precultured individually overnight at 35 °C. Using a 10 µL loop, each diluted preculture was plated on MH impregnated with antibiotic at a concentration 4X the highest MIC tested in the P-AST well exhibiting resistance. After incubating 12–18 h at 35 °C, any growth was interpreted as confirming the resistant phenotype.

### 4.8. Analysis Workflow

**Figure 1:**
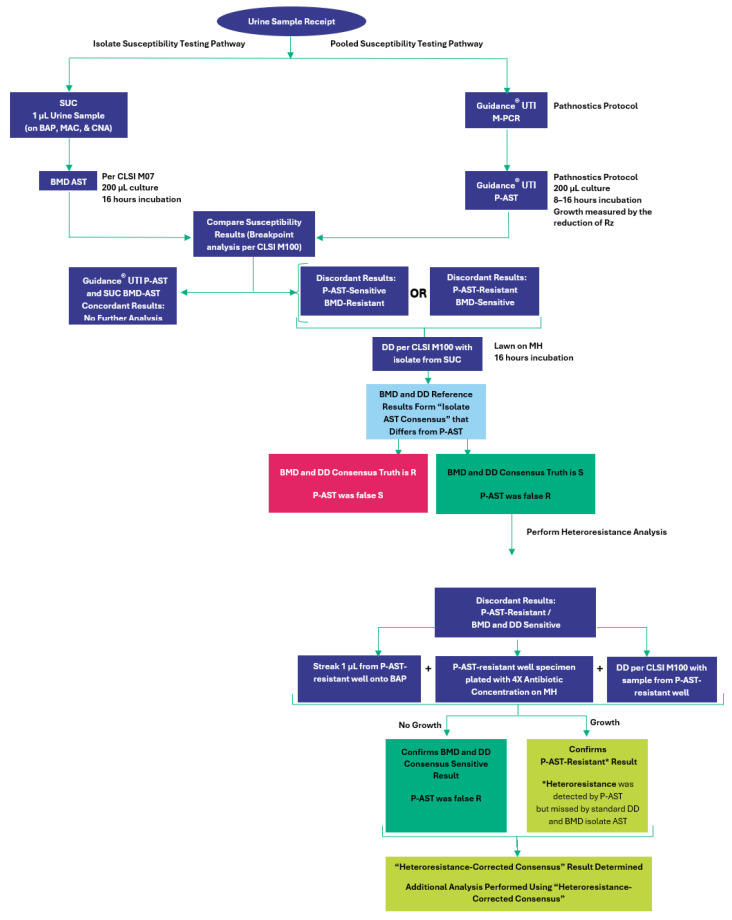
Overview of study workflow.SUC = standard urine culture; BAP = blood agar plate; MAC = MacConkey agar plate; CNA = Columbia nalidixic acid agar plate; BMD = microbroth dilution; DD = disk diffusion; AST = antibiotic susceptibility testing; CLSI = Clinical and Laboratory Standards Institute; MH = Mueller–Hinton agar; M-PCR = multiplex polymerase chain reaction; P-AST = pooled antibiotic susceptibility testing; Rz = resazurin.

Upon receipt, the urine samples were subjected, in parallel, to standard urine culture and M-PCR for microbial identification and quantification. Specimens with a single organism detected by M-PCR were chosen for inclusion in the study. Specimens were excluded if the SUC results were negative for microbial detection, “mixed flora” or “contaminated”, or positive detection of multiple species (polymicrobial). Antibiotic susceptibility testing was conducted on these samples. Isolated colonies from SUC were tested by BMD, while P-AST was conducted as described in the Methods section. The susceptibility results were then compared between the two techniques. Cases in which the susceptibility results were discrepant between the two techniques underwent DD AST using the isolates from SUC to resolve the discrepancy (Figure 1).

If the BMD and DD results were in agreement, the result was defined as “Isolate AST Consensus”. If BMD and DD were conflicting, no susceptibility “Isolate AST Consensus” was determined, and the sample was excluded from the study. Natural technical variability of these two standardized isolate AST techniques sometimes results in such discrepancies [57,58,59]. For specimens with P-AST-sensitive and BMD/DD consensus-resistant discrepant results, no further testing was performed. If P-AST was resistant and the BMD/DD consensus was sensitive, the specimen was worked up for heteroresistance as described below.

### 4.9. Analysis Workflow for Detecting Heteroresistant Phenotypes

For specimens with P-AST-resistant and BMD/DD consensus-susceptible results, 1 µL from the resistant P-AST culture well was plated onto a BAP. Simultaneously, a specimen from the resistant P-AST well was plated with 4X antibiotic concentration on MH and subjected to DD AST. If no growth was observed, the “Isolate AST Consensus” was affirmed as sensitive, and the P-AST results were deemed falsely resistant. If growth was observed in DD, BMD, or both, the resistant P-AST result was affirmed, and the BMD/DD initial “Isolate AST Consensus” result determination was deemed falsely sensitive due to heteroresistance. These were cases in which heteroresistance was detected by the P-AST technique and missed by the standard isolate AST techniques, BAP and DD. The “Heteroresistance-Corrected Consensus” in the Results reflect the heteroresistant-corrected analysis. Both analyses with and without accounting for heteroresistance are included in the results.

### 4.10. Statistical Analyses

The metrics of P-AST validation were calculated according to CLSI standards [52]. Essential agreement (EA%) = Number of tests with minimum inhibitory concentration (MIC) within ± one two-fold dilution/total tests × 100. Categorical agreement (CA%) = Number of tests with same category results/total tests × 100. Very major errors (VME%) = Number of tests where the P-AST result is “S” and the “Consensus” result is “R”/total tests × 100. Major errors (ME%) = Number of tests where the P-AST result is “R” and the “Consensus” result is “S”/total tests × 100. Minor errors (mE%) = % of tests where 1) the P-AST result is “I” and the “Consensus” result is either “S” or “R” OR 2) the P-AST result is either “S” or “R” and the “Consensus” result is “I”. In the heteroresistance-corrected analysis, cases that showed heteroresistance were categorized as VMEs for BMD and DD AST instead of MEs by P-AST, as the resistance subpopulation was missed by the standard techniques and detected by P-AST. For all the measures, 95% confidence intervals were calculated using the Agresti–Coull method. All statistical calculations were performed using Python 3.10.12.

## 5. Conclusions

P-AST, a component of the Guidance^®^ UTI assay, which rapidly measures the antibiotic susceptibility of a bacterial pellet of cultivable organisms from a urine specimen, demonstrates high essential agreement (≥90%) and low rates of very major (<1.5%) and major errors (<3%) within the thresholds established by CLSI for AST testing. Minor errors (<10%) and categorical agreement (>88%) were also within these thresholds. P-AST also demonstrated the capacity to detect heteroresistance phenotypes, which further reduced P-AST major error rates and demonstrated potential very major errors for standard methods. These results were demonstrated in monomicrobial samples, where an identical pathogen was identified by Guidance UTI and the standard culture methods, with future work in process to evaluate polymicrobial samples.

## Figures and Tables

**Table 1:** ICD-10-CM codes.

ICD-10-CM Code	Description	*n* (%)
N39.0	Urinary tract infection, site not specified	191 (76.4%)
R30.0	Dysuria	29 (11.6%)
R82.998	Other abnormal findings in urine	15 (6.0%)
R35.0	Frequency of micturition	13 (5.2%)
Z87.440	Personal history of urinary (tract) infections	13 (5.2%)
Other	NA	63 (25.2%)

**Table 2:** Monomicrobial organism detection frequency among all included specimens.

Stain	Monomicrobial Organism Detected	*n*	%
Gram-Negative	*Citrobacter freundii*	3	1.2%
	Enterobacter Group	8	3.2%
	*Escherichia coli*	126	50.4%
	*Klebsiella oxytoca*	2	0.8%
	*Klebsiella pneumoniae*	32	12.8%
	*Morganella morganii*	4	1.6%
	*Pseudomonas aeruginosa*	11	4.4%
	*Proteus mirabilis*	13	5.2%
	*Serratia marcescens*	1	0.4%
Gram-Positive	Coagulase-negative Staph group	10	4.0%
	*Enterococcus faecalis*	37	14.8%
	*Staphylococcus aureus*	3	1.2%

**Table 3:** P-AST performance contingency table for all monomicrobial cases.

	P-AST Sensitive	P-AST Intermediate	P-AST Resistant	Total
Isolate AST Consensus Sensitive	2459 (53.5%)	103 (2.2%)	100 (2.2%)	2662 (57.9%)
Isolate AST Consensus Intermediate	137 (3.0%)	65 (1.4%)	88 (1.9%)	290 (5.2%)
Isolate AST Consensus Resistant	32 (0.7%)	87 (1.9%)	1523 (33.2%)	1642 (35.7%)
Total	2628 (57.2%)	255 (5.6%)	1711 (37.2%)	4594 (100.0%)

P-AST = pooled antibiotic susceptibility testing. AST = antibiotic susceptibility testing. Background color indicates instances where both methods had concordant results.

**Table 4:** P-AST versus isolate AST MBD performance: all organism–antibiotic combinations.

Essential Agreement (EA) % (95% CI), *n* = 4368	95.1% (94.4%, 95.7%)
Very Major Errors (VMEs) % (95% CI) *n* = 32	0.7% (0.5%, 1.0%)
Major Errors (MEs) % (95% CI) *n* = 100	2.2% (1.8%, 2.6%)
Minor Errors (mEs) % (95% CI) *n* = 415	9.0% (8.2%, 9.9%)
Minor Errors with Essential Agreement % (95% CI) n = 321	7.0% (6.3%, 7.8%)
Categorical Agreement (CA) % (95% CI) *n* = 4047	88.1% (87.1%, 89.0%)

EA% describes the agreement between MIC values. VMEs are also known as false-susceptible errors. MEs are also known as false-resistant errors. mEs are discrepancies between categorical calls involving an intermediate “I” call by either method. CA% describes the agreement between the susceptible, intermediate, and resistant calls. Details of these measures and their calculations are in the statistical analyses subsection of the Materials and Methods section.

**Table 5:** P-AST performance contingency table for all monomicrobial *E. coli* cases.

	P-AST Sensitive	P-AST Intermediate	P-AST Resistant	Total
Isolate AST Consensus Sensitive	1418 (61.0%)	43 (1.8%)	49 (2.1%)	1510 (64.9%)
Isolate AST Consensus Intermediate	66 (2.8%)	26 (1.1%)	19 (0.8%)	111 (3.8%)
Isolate AST Consensus Resistant	11 (0.5%)	44 (1.9%)	650 (27.9%)	705 (30.3%)
Total	1495 (64.3%)	113 (4.9%)	718 (30.9%)	2326 (100%)

P-AST = pooled antibiotic susceptibility testing. AST = antibiotic susceptibility testing. Background color indicates instances where both methods had concordant results.

**Table 6:** P-AST versus isolate AST performance: all monomicrobial *E. coli*–antibiotic combinations.

Essential Agreement (EA) % (95% CI) *n* = 2231	95.9% (95.0%, 96.7%)
Very Major Errors (VMEs) % (95% CI) *n* = 11	0.5% (0.3%, 0.9%)
Major Errors (MEs) % (95% CI) *n* = 49	2.1% (1.6%, 2.8%)
Minor Errors (mEs) % (95% CI) *n* = 172	7.4% (6.4%, 8.5%)
Minor Errors with Essential Agreement % (95% CI) *n* = 139	6.0% (5.1%, 7.0%)
Categorical Agreement (CA) % (95% CI) *n* = 2094	90.0% (88.7%, 91.2%)

EA% describes the agreement between MIC values. VMEs are also known as false-susceptible errors. MEs are also known as false-resistant errors. mEs are discrepancies between categorical calls involving an intermediate “I” call by either method. CA% describes the agreement between the susceptible, intermediate, and resistant calls. Details of these measures and their calculations are in the statistical analyses subsection of the Materials and Methods section.

**Table 7:** P-AST performance contingency table for all non-*E. coli* monomicrobial cases.

	P-AST Sensitive	P-AST Intermediate	P-AST Resistant	Total
Isolate AST Consensus Sensitive	1041 (45.9%)	60 (2.6%)	51 (2.2%)	1152 (50.8%)
Isolate AST Consensus Intermediate	71 (3.1%)	39 (1.7%)	69 (3.0%)	179 (6.7%)
Isolate AST Consensus Resistant	21 (0.9%)	43 (1.9%)	873 (38.5%)	937 (41.3%)
Total	1133 (50.0%)	142 (6.3%)	993 (43.8%)	2268 (100.0%)

P-AST = pooled antibiotic susceptibility testing. AST = antibiotic susceptibility testing. Background color indicates instances where both methods had concordant results.

**Table 8:** P-AST versus isolate AST performance: all non-*E. coli* monomicrobial organism–antibiotic combinations.

Essential Agreement (EA) % (95% CI) *n* = 2137	94.2% (93.2%, 95.1%)
Very Major Errors (VMEs) % (95% CI) *n* = 21	0.9% (0.6%, 1.4%)
Major Errors (MEs) % (95% CI) *n* = 51	2.2% (1.7%, 2.9%)
Minor Errors (mEs) % (95% CI) *n* = 243	10.7% (9.5%, 12.1%)
Minor Errors with Essential Agreement % (95% CI) *n* = 182	8.0% (7.0%, 9.2%)
Categorical Agreement (CA) % (95% CI) *n* = 1953	86.1% (84.6%, 87.5%)

EA% describes the agreement between MIC values. VMEs are also known as false-susceptible errors. MEs are also known as false-resistant errors. mEs are discrepancies between categorical calls involving an intermediate “I” call by either method. CA% describes the agreement between the susceptible, intermediate, and resistant calls. Details of these measures and their calculations are in the statistical analyses subsection of the Materials and Methods section.

**Table 9:** P-AST performance contingency table for all cases with monomicrobial Gram-negative organisms.

	P-AST Sensitive	P-AST Intermediate	P-AST Resistant	Total
Isolate AST Consensus Sensitive	2110 (57.4%)	86 (2.3%)	83 (2.3%)	2279 (62.0%)
Isolate AST Consensus Intermediate	114 (3.1%)	46 (1.3%)	38 (1.0%)	198 (4.4%)
Isolate AST Consensus Resistant	28 (0.8%)	78 (2.1%)	1094 (29.8%)	1200 (32.6%)
Total	2252 (61.2%)	210 (5.7%)	1215 (33.0%)	3677 (100.0%)

P-AST = pooled antibiotic susceptibility testing. AST = antibiotic susceptibility testing. Background color indicates instances where both methods had concordant results.

**Table 10:** P-AST versus isolate AST performance: all cases with monomicrobial Gram-negative organisms.

Essential Agreement (EA) % (95% CI) *n* = 3511	95.5% (94.8%, 96.1%)
Very Major Errors (VMEs) % (95% CI) *n* = 28	0.8% (0.5%, 1.1%)
Major Errors (MEs) % (95% CI) *n* = 83	2.3% (1.8%, 2.8%)
Minor Errors (mEs) % (95% CI) *n* = 316	8.6% (7.7%, 9.5%)
Minor Errors with Essential Agreement % (95% CI) *n* = 261	7.1% (6.3%, 8.0%)
Categorical Agreement (CA) % (95% CI) *n* = 3250	88.4% (87.3%, 89.4%)

EA% describes the agreement between MIC values. VMEs are also known as false-susceptible errors. MEs are also known as false-resistant errors. mEs are discrepancies between categorical calls involving an intermediate “I” call by either method. CA% describes the agreement between the susceptible, intermediate, and resistant calls. Details of these measures and their calculations are in the statistical analyses subsection of the Materials and Methods section.

**Table 11:** P-AST performance contingency table for all cases with monomicrobial Gram-negative organisms, excluding *E. coli*.

	P-AST Sensitive	P-AST Intermediate	P-AST Resistant	Total
Isolate AST Consensus Sensitive	692 (51.2%)	43 (3.2%)	34 (2.5%)	769 (56.9%)
Isolate AST Consensus Intermediate	48 (3.6%)	20 (1.5%)	19 (1.4%)	87 (5.4%)
Isolate AST Consensus Resistant	17 (1.3%)	34 (2.5%)	444 (32.9%)	495 (36.6%)
Total	757 (56.0%)	97 (7.2%)	497 (36.8%)	1351 (100.0%)

P-AST = pooled antibiotic susceptibility testing. AST = antibiotic susceptibility testing. Background color indicates instances where both methods had concordant results.

**Table 12:** P-AST versus isolate AST performance: all cases with monomicrobial Gram-negative organisms, excluding *E. coli*.

Essential Agreement (EA) % (95% CI) *n* = 1280	94.7% (93.4%, 95.8%)
Very Major Errors (VMEs) % (95% CI) *n* = 17	1.3% (0.8%, 2.0%)
Major Errors (MEs) % (95% CI) *n* = 34	2.5% (1.8%, 3.5%)
Minor Errors (mEs) % (95% CI) *n* = 144	10.7% (9.1%, 12.4%)
Minor Errors with Essential Agreement % (95% CI) *n* = 122	9.0% (7.6%, 10.7%)
Categorical Agreement (CA) % (95% CI) *n* = 1156	85.6% (83.6%, 87.3%)

EA% describes the agreement between MIC values. VMEs are also known as false-susceptible errors. MEs are also known as false-resistant errors. mEs are discrepancies between categorical calls involving an intermediate “I” call by either method. CA% describes the agreement between the susceptible, intermediate, and resistant calls. Details of these measures and their calculations are in the statistical analyses subsection of the Materials and Methods section.

**Table 13:** P-AST performance contingency table for all cases with monomicrobial Gram-positive organisms.

	P-AST Sensitive	P-AST Intermediate	P-AST Resistant	Total
Isolate AST Consensus Sensitive	349 (38.1%)	17 (1.9%)	17 (1.9%)	383 (41.8%)
Isolate AST Consensus Intermediate	23 (2.5%)	19 (2.1%)	50 (5.5%)	92 (8.5%)
Isolate AST Consensus Resistant	4 (0.4%)	9 (1.0%)	429 (46.8%)	442 (48.2%)
Total	376 (41.0%)	45 (4.9%)	496 (54.1%)	917 (100.0%)

P-AST = pooled antibiotic susceptibility testing. AST = antibiotic susceptibility testing. Background color indicates instances where both methods had concordant results.

**Table 14:** P-AST versus isolate AST performance: all cases with monomicrobial Gram-positive organisms.

Essential Agreement (EA) % (95% CI) *n* = 857	93.5% (91.7%, 94.9%)
Very Major Errors (VMEs) % (95% CI) *n* = 4	0.4% (0.1%, 1.2%)
Major Errors (MEs) % (95% CI) *n* = 17	1.9% (1.1%, 3.0%)
Minor Errors (mEs) % (95% CI) *n* = 99	10.8% (8.9%, 13.0%)
Minor Errors with Essential Agreement % (95% CI) *n* = 60	6.5% (5.1%, 8.3%)
Categorical Agreement (CA) % (95% CI) *n* = 797	86.9% (84.6%, 88.9%)

EA% describes the agreement between MIC values. VMEs are also known as false-susceptible errors. MEs are also known as false-resistant errors. mEs are discrepancies between categorical calls involving an intermediate “I” call by either method. CA% describes the agreement between the susceptible, intermediate, and resistant calls. Details of these measures and their calculations are in the statistical analyses subsection of the Materials and Methods section.

**Table 15:** (**a**) P-AST performance contingency table for all monomicrobial cases after correction for heteroresistance. (**b**) Isolate BMD AST performance contingency table for all monomicrobial cases after correction for heteroresistance.

**(a)**				
	**P-AST** **Sensitive**	**P-AST** **Intermediate**	**P-AST** **Resistant**	**Total**
Heteroresistance-Corrected Consensus Sensitive	2459 (53.5%)	103 (2.2%)	35 (0.8%)	2597 (56.5%)
Heteroresistance-Corrected Consensus Intermediate	137 (3.0%)	65 (1.4%)	88 (1.9%)	290 (5.2%)
Heteroresistance-Corrected Consensus Resistant	32 (0.7%)	87 (1.9%)	1588 (34.6%)	1707 (37.2%)
Total	2628 (57.2%)	255 (5.6%)	1711 (37.2%)	4594 (100.0%)
**(b)**				
	**BMD AST** **Sensitive**	**BMD AST** **Intermediate**	**BMD AST** **Resistant**	**Total**
Heteroresistance-Corrected Consensus Sensitive	2597 (56.5%)	0 (0.0%)	0 (0.0%)	2597 (56.5%)
Heteroresistance-Corrected Consensus Intermediate	0 (0.0%)	290 (6.3%)	0 (0.0%)	290 (6.3%)
Heteroresistance-Corrected Consensus Resistant	65 (1.4%)	0 (0.0%)	1642 (35.7%)	1707 (37.2%)
Total	2662 (57.9%)	290 (6.3%)	1642 (35.7%)	4594 (100.0%)

P-AST = pooled antibiotic susceptibility testing. AST = antibiotic susceptibility testing. BMD = broth microdilution. Background color indicates instances where both methods had concordant results.

**Table 16:** P-AST heteroresistance-corrected consensus versus isolate AST performance: all organism–antibiotic combinations.

	P-AST		Isolate BMD AST	
	*n*	% (95% CI)	*n*	% (95% CI)
Very Major Errors (VMEs)	32	0.7% (0.5%, 1.0%)	65	1.4% (1.1%, 1.8%)
Major Errors (MEs)	35	0.8% (0.5%, 1.1%)	0	0.0% (0.0%, 0.1%)
Minor Errors (mEs)	415	9.0% (8.2%, 9.9%)	0	0.0% (0.0%, 0.1%)

VMEs are also known as false-susceptible errors. MEs are also known as false-resistant errors. mEs are discrepancies between categorical calls involving an intermediate “I” call by either method. Details of these measures and their calculations are in the statistical analyses subsection of the Materials and Methods section.

**Table 17:** Matrix of relative heteroresistance rates by organism–antibiotic combination.

	Amoxicillin/Clavulanate	Ampicillin	Ampicillin/Sulbactam	Cefaclor	Cefazolin	Cefepime	Cefoxitin	Ceftazidime	Ceftriaxone	Ciprofloxacin	Fosfomycin	Gentamicin	Levofloxacin	Meropenem	Nitrofurantoin	Piperacillin/Tazobactam	Tetracycline	Trimethoprim/Sulfamethoxazole	Vancomycin
*C. freundii*																			
CoNS																			
Enterobacter Group						12.5%		33.3%	14.3%					25.0%			16.7%		
*E. faecalis*													2.8%				2.8%		10.8%
*E. coli*	0.8%	3.2%	0.8%	2.7%	3.3%	2.4%	3.3%		2.5%	2.4%			2.4%	2.4%	0.8%		0.8%	0.8%	
*K. oxytoca*																			
*K. pneumoniae*	3.2%			3.4%	6.5%	3.2%	3.1%	3.2%	3.1%					3.1%		3.1%	3.2%	3.1%	
*M. morganii*																			
*P. mirabilis*		7.7%																	
*P. aeruginosa*								10.0%					9.1%	9.1%		9.1%			
*S. marcescens*																			
*S. aureus*																			

Organisms are plotted down the left side, and antibiotics are plotted across the top, with the relative frequency of heteroresistance (% of heteroresistant results out of all the test results for that organism–antibiotic combination) plotted at the intersection. Empty cells indicate that no heteroresistance was observed for that combination. CoNS = Coagulase-negative Staphylococcus and includes *S. epidermidis*, *S. haemolyticus*, *S. lugdunensis*, and *S. saprophyticus*.

## Data Availability

The original data presented in the study are openly available in FigShare at https://doi.org/10.6084/m9.figshare.27324660.v1.

## Supplementary Materials

The following supporting information can be downloaded at: https://www.mdpi.com/article/10.3390/antibiotics13121214/s1. Supplemental Table S1. Microorganism Characteristics.

## References

[1] Antimicrobial Resistance Facts and Stats|Antimicrobial Resistance|CDC. https://www.cdc.gov/antimicrobial-resistance/data-research/facts-stats/index.html.

[2] Li X., Fan H., Zi H., Hu H., Li B., Huang J., Luo P., Zeng X. (2022). Global and Regional Burden of Bacterial Antimicrobial Resistance in Urinary Tract Infections in 2019. J. Clin. Med..

[3] Wolfe A.J., Brubaker L. (2015). “Sterile Urine” and the Presence of Bacteria. Eur. Urol..

[4] Brubaker L., Chai T.C., Horsley H., Khasriya R., Moreland R.B., Wolfe A.J. (2023). Tarnished Gold—The “Standard” Urine Culture: Reassessing the Characteristics of a Criterion Standard for Detecting Urinary Microbes. Front. Urol..

[5] Price T.K., Dune T., Hilt E.E., Thomas-White K.J., Kliethermes S., Brincat C., Brubaker L., Wolfe A.J., Mueller E.R., Schreckenberger P.C. (2016). The Clinical Urine Culture: Enhanced Techniques Improve Detection of Clinically Relevant Microorganisms. J. Clin. Microbiol..

[6] Price T.K., Hilt E.E., Dune T.J., Mueller E.R., Wolfe A.J., Brubaker L. (2018). Urine Trouble: Should We Think Differently about UTI?. Int. Urogynecol J..

[7] Wang D., Haley E., Luke N., Mathur M., Festa R., Zhao X., Anderson L.A., Allison J.L., Stebbins K.L., Diaz M.J. (2023). Emerging and Fastidious Uropathogens Were Detected by M-PCR with Similar Prevalence and Cell Density in Catheter and Midstream Voided Urine Indicating the Importance of These Microbes in Causing UTIs. Infect. Drug Resist..

[8] Haley E., Luke N., Mathur M., Festa R.A., Wang J., Jiang Y., Anderson L.A., Baunoch D. (2024). The Prevalence and Association of Different Uropathogens Detected by M-PCR with Infection-Associated Urine Biomarkers in Urinary Tract Infections. Res. Rep. Urol..

[9] Kaprou G.D., Bergšpica I., Alexa E.A., Alvarez-Ordóñez A., Prieto M. (2021). Rapid Methods for Antimicrobial Resistance Diagnostics. Antibiotics.

[10] Baunoch D., Luke N., Wang D., Vollstedt A., Zhao X., Ko D.S.C., Huang S., Cacdac P., Sirls L.T. (2021). Concordance Between Antibiotic Resistance Genes and Susceptibility in Symptomatic Urinary Tract Infections. Infect. Drug Resist..

[11] Bard J., Lee F. (2018). Why Can’t We Just Use PCR? The Role of Genotypic versus Phenotypic Testing for Antimicrobial Resistance Testing. Clin. Microbiol. Newsl..

[12] Simner P.J., Rauch C.A., Martin I.W., Sullivan K.V., Rhoads D., Rolf R., She R., Souers R.J., Wojewoda C., Humphries R.M. (2022). Raising the Bar: Improving Antimicrobial Resistance Detection by Clinical Laboratories by Ensuring Use of Current Breakpoints. Open Forum Infect. Dis..

[13] Gajic I., Kabic J., Kekic D., Jovicevic M., Milenkovic M., Culafic D.M., Trudic A., Ranin L., Opavski N. (2022). Antimicrobial Susceptibility Testing: A Comprehensive Review of Currently Used Methods. Antibiotics.

[14] Antimicrobial Susceptibility Test Breakpoint Updates: Challenges and Considerations for Laboratory Validation|Medical Laboratory Observer. https://www.mlo-online.com/diagnostics/microbiology/article/21283829/antimicrobial-susceptibility-test-breakpoint-updates-challenges-and-considerations-for-laboratory-validation.

[15] Nicoloff H., Hjort K., Levin B.R., Andersson D.I. (2019). The High Prevalence of Antibiotic Heteroresistance in Pathogenic Bacteria Is Mainly Caused by Gene Amplification. Nat. Microbiol..

[16] (2024). Performance Standards for Antimicrobial Susceptibility Testing Guideline M100.

[17] Haley E., Luke N., Korman H., Baunoch D., Wang D., Zhao X., Mathur M. (2023). Improving Patient Outcomes While Reducing Empirical Treatment with Multiplex-Polymerase-Chain-Reaction/Pooled-Antibiotic-Susceptibility-Testing Assay for Complicated and Recurrent Urinary Tract Infections. Diagnostics.

[18] Korman H.J., Baunoch D., Luke N., Wang D., Zhao X., Levin M., Wenzler D.L., Mathur M. (2023). A Diagnostic Test Combining Molecular Testing with Phenotypic Pooled Antibiotic Susceptibility Improved the Clinical Outcomes of Patients with Non-E. Coli or Polymicrobial Complicated Urinary Tract Infections. Res. Rep. Urol..

[19] Akhlaghpour M., Haley E., Parnell L., Luke N., Mathur M., Festa R.A., Percaccio M., Magallon J., Remedios-Chan M., Rosas A. (2024). Urine Biomarkers Individually and as a Consensus Model Show High Sensitivity and Specificity for Detecting UTIs. BMC Infect. Dis..

[20] Döpfer D., Buist W., Soyer Y., Munoz M.A., Zadoks R.N., Geue L., Engel B. (2008). Assessing Genetic Heterogeneity within Bacterial Species Isolated from Gastrointestinal and Environmental Samples: How Many Isolates Does It Take?. Appl. Environ. Microbiol..

[21] Singh B.R., Sinha D.K., Jayakumar V., Agri H., Yadav A., Karthikeyan R. (2023). Effect of Picking Multiple Colonies on Antimicrobial Susceptibility Diagnostic Outcome in a Clinical Bacteriology Setting. Infect. Dis. Res..

[22] Maciel J.F., Gressler L.T., da Silveira B.P., Dotto E., Balzan C., Matter L.B., Siqueira F.M., Vargas A.P.C. (2020). de Caution at Choosing a Particular Colony-forming Unit from Faecal Escherichia Coli: It May Not Represent the Sample Profile. Lett. Appl. Microbiol..

[23] Wagenlehner F.M., Lichtenstern C., Rolfes C., Mayer K., Uhle F., Weidner W., Weigand M.A. (2013). Diagnosis and Management for Urosepsis. Int. J. Urol..

[24] Festa R.A., Luke N., Mathur M., Parnell L., Wang D., Zhao X., Magallon J., Remedios-Chan M., Nguyen J., Cho T. (2023). A Test Combining Multiplex-PCR with Pooled Antibiotic Susceptibility Testing Has High Correlation with Expanded Urine Culture for Detection of Live Bacteria in Urine Samples of Suspected UTI Patients. Diagn. Microbiol. Infect. Dis..

[25] Haley E., Luke N., Mathur M., Festa R.A., Wang J., Jiang Y., Anderson L., Baunoch D. (2023). Comparison Shows That Multiplex Polymerase Chain Reaction Identifies Infection-Associated Urinary Biomarker–Positive Urinary Tract Infections That Are Missed by Standard Urine Culture. Eur. Urol. Open Sci..

[26] Andersson D.I., Nicoloff H., Hjort K. (2019). Mechanisms and Clinical Relevance of Bacterial Heteroresistance. Nat. Rev. Microbiol..

[27] Bobay L.-M., Traverse C.C., Ochman H. (2015). Impermanence of Bacterial Clones. Proc. Natl. Acad. Sci. USA.

[28] Levin B.R., Berryhill B.A., Gil-Gil T., Manuel J.A., Smith A.P., Choby J.E., Andersson D.I., Weiss D.S., Baquero F. (2024). Theoretical Considerations and Empirical Predictions of the Pharmaco- and Population Dynamics of Heteroresistance. Proc. Natl. Acad. Sci. USA.

[29] Casadesús J., Low D.A. (2013). Programmed Heterogeneity: Epigenetic Mechanisms in Bacteria. J. Biol. Chem..

[30] Seib K.L., Srikhanta Y.N., Atack J.M., Jennings M.P. (2020). Epigenetic Regulation of Virulence and Immunoevasion by Phase-Variable Restriction-Modification Systems in Bacterial Pathogens. Annu. Rev. Microbiol..

[31] Adam M., Murali B., Glenn N.O., Potter S.S. (2008). Epigenetic Inheritance Based Evolution of Antibiotic Resistance in Bacteria. BMC Evol. Biol..

[32] Pereira C., Larsson J., Hjort K., Elf J., Andersson D.I. (2021). The Highly Dynamic Nature of Bacterial Heteroresistance Impairs Its Clinical Detection. Commun. Biol..

[33] Saito M., Katayama Y., Hishinuma T., Iwamoto A., Aiba Y., Kuwahara-Arai K., Cui L., Matsuo M., Aritaka N., Hiramatsu K. (2014). “Slow VISA,” a Novel Phenotype of Vancomycin Resistance, Found In Vitro in Heterogeneous Vancomycin-Intermediate Staphylococcus Aureus Strain Mu3. Antimicrob. Agents Chemother..

[34] Band V.I., Weiss D.S. (2021). Heteroresistance to Beta-Lactam Antibiotics May Often Be a Stage in the Progression to Antibiotic Resistance. PLoS Biol..

[35] Band V.I., Weiss D.S. (2019). Heteroresistance: A Cause of Unexplained Antibiotic Treatment Failure?. PLoS Pathog..

[36] Band V.I., Crispell E.K., Napier B.A., Herrera C.M., Tharp G.K., Vavikolanu K., Pohl J., Read T.D., Bosinger S.E., Trent M.S. (2016). Antibiotic Failure Mediated by a Resistant Subpopulation in Enterobacter Cloacae. Nat. Microbiol..

[37] Antibiotic Heteroresistance: What Is It and How Does It Impact Patients?. https://www.contagionlive.com/view/antibiotic-heteroresistance-what-is-it-and-how-does-it-impact-patients-.

[38] Tetz G., Tetz V. (2022). Overcoming Antibiotic Resistance with Novel Paradigms of Antibiotic Selection. Microorganisms.

[39] Mouratidou A., Karbach J., d’Hoedt B., Al-Nawas B. (2011). Antibiotic Susceptibility of Cocultures in Polymicrobial Infections Such as Peri-Implantitis or Periodontitis: An In Vitro Model. J. Periodontol..

[40] Ghuneim L.-A.J., Raghuvanshi R., Neugebauer K.A., Guzior D.V., Christian M.H., Schena B., Feiner J.M., Castillo-Bahena A., Mielke J., McClelland M. (2022). Complex and Unexpected Outcomes of Antibiotic Therapy against a Polymicrobial Infection. ISME J..

[41] Sorg R.A., Lin L., van Doorn G.S., Sorg M., Olson J., Nizet V., Veening J.-W. (2016). Collective Resistance in Microbial Communities by Intracellular Antibiotic Deactivation. PLoS Biol..

[42] Meredith H.R., Srimani J.K., Lee A.J., Lopatkin A.J., You L. (2015). Collective Antibiotic Tolerance: Mechanisms, Dynamics and Intervention. Nat. Chem. Biol..

[43] Vega N.M., Gore J. (2014). Collective Antibiotic Resistance: Mechanisms and Implications. Curr. Opin. Microbiol..

[44] Little W., Black C., Smith A.C. (2021). Clinical Implications of Polymicrobial Synergism Effects on Antimicrobial Susceptibility. Pathogens.

[45] Denk-Lobnig M., Wood K.B. (2023). Antibiotic Resistance in Bacterial Communities. Curr. Opin. Microbiol..

[46] De Vos M.G., Zagorski M., McNally A., Bollenbach T. (2017). Interaction Networks, Ecological Stability, and Collective Antibiotic Tolerance in Polymicrobial Infections. Proc. Natl. Acad. Sci. USA.

[47] Bottery M.J., Matthews J.L., Wood A.J., Johansen H.K., Pitchford J.W., Friman V.-P. (2021). Inter-Species Interactions Alter Antibiotic Efficacy in Bacterial Communities. Isme J..

[48] Daly A., Baunoch D., Rehling K., Luke N., Campbell M., Cacdac P., Penaranda M., Opel M., Huang S., Zhao X. (2020). Utilization of M-PCR and P-AST for Diagnosis and Management of Urinary Tract Infections in Home-Based Primary Care. JOJ Uro Nephron.

[49] Hilt E.E., McKinley K., Pearce M.M., Rosenfeld A.B., Zilliox M.J., Mueller E.R., Brubaker L., Gai X., Wolfe A.J., Schreckenberger P.C. (2014). Urine Is Not Sterile: Use of Enhanced Urine Culture Techniques To Detect Resident Bacterial Flora in the Adult Female Bladder. J. Clin. Microbiol..

[50] Older Persons|UNHCR. https://emergency.unhcr.org/protection/persons-risk/older-persons.

[51] McCann E., Sung A.H., Ye G., Vankeepuram L., Tabak Y.P. (2020). Contributing Factors to the Clinical and Economic Burden of Patients with Laboratory-Confirmed Carbapenem-Nonsusceptible Gram-Negative Urinary Tract Infections. Clin. Outcomes Res..

[52] CLSI Breakpoint Implementation Toolkit 2023. https://clsi.org/media/3k3idx3b/part_c_breakpoint_implementation_summary.pdf.

[53] Weinstein M.P., Lewis J.S. (2020). The Clinical and Laboratory Standards Institute Subcommittee on Antimicrobial Susceptibility Testing: Background, Organization, Functions, and Processes. J. Clin. Microbiol..

[54] Guidance on Review Criteria for Assessment of Antimicrobial Devices. https://www.fda.gov/files/Guidance-for-Industry-and-FDA--Class-II-Special-Controls-Guidance-Document--Antimicrobial-Susceptibility-Test-%28AST%29-Systems.pdf.

[55] Wojno K.J., Baunoch D., Luke N., Opel M., Korman H., Kelly C., Jafri S.M.A., Keating P., Hazelton D., Hindu S. (2019). Multiplex PCR Based Urinary Tract Infection (UTI) Analysis Compared to Traditional Urine Culture in Identifying Significant Pathogens in Symptomatic Patients. Urology.

[56] (2024). Methods for Dilution and Antimicrobial Susceptibility Tests for Bacteria That Grow Aerobically Guideline M07.

[57] Jenkins S.G., Jerris R.C. (2011). Critical Assessment of Issues Applicable to Development of Antimicrobial Susceptibility Testing Breakpoints. J. Clin. Microbiol..

[58] Hombach M., Ochoa C., Maurer F.P., Pfiffner T., Böttger E.C., Furrer R. (2016). Relative Contribution of Biological Variation and Technical Variables to Zone Diameter Variations of Disc Diffusion Susceptibility Testing. J. Antimicrob. Chemother..

[59] Bonnin R.A., Emeraud C., Jousset A.B., Naas T., Dortet L. (2022). Comparison of Disk Diffusion, MIC Test Strip and Broth Microdilution Methods for Cefiderocol Susceptibility Testing on Carbapenem-Resistant Enterobacterales. Clin. Microbiol. Infect..

