# Supplementary material for: Pooled Antibiotic Susceptibility Testing Performs Within CLSI Standards for Validation When Measured Against Broth Microdilution and Disk Diffusion Antibiotic Susceptibility Testing of Cultured Isolates
Source: Antibiotics (Basel). 2024 Dec 14;13(12):1214. doi: 10.3390/antibiotics13121214 (PMC11672409; doi:10.3390/antibiotics13121214)
Supplement: Supplementary file 1 [file antibiotics-13-01214-s001.zip › antibiotics-3333361-supplementary.pdf]

Supplemental Table S1. Microorganism Charachteristics

Microorganism	Bacterial Cell Wall (gram-negative[a] or gram-positive[a])	Growth (fastidious[b] or non-fastidious[b])	P-AST provides appropriate growth conditions for organism	Classification (classical[c] or emerging[c])
Acinetobacter baumannii	gram-negative	non-fastidious	Yes	emerging
Actinotignum schaalii	gram-positive	fastidious	No	emerging
Aerococcus urinae	gram-positive	fastidious	No	emerging
Alloscardovia omnicolens	gram-positive	fastidious	No	emerging
Candida albicans	NA - yeast	NA - yeast	No	classical
Candida auris	NA - yeast	NA - yeast	No	classical
Candida glabrata	NA - yeast	NA - yeast	No	classical
Candida parapsilosis	NA - yeast	NA - yeast	No	classical
Citrobacter freundii	gram-negative	non-fastidious	Yes	classical
Citrobacter koseri	gram-negative	non-fastidious	Yes	classical
Corynebacterium riegelii	gram-positive	fastidious	No	emerging
Enterococcus faecalis	gram-positive	non-fastidious	Yes	classical
Enterococcus faecium	gram-positive	fastidious	No	classical
Escherichia coli	gram-negative	non-fastidious	Yes	classical
Gardnerella vaginalis	gram-positive	fastidious	No	emerging
Klebsiella oxytoca	gram-negative	non-fastidious	Yes	classical
Klebsiella pneumoniae	gram-negative	non-fastidious	Yes	classical
Morganella morganii	gram-negative	non-fastidious	Yes	classical
Mycoplasma hominis	NA - no cell wall	fastidious	No	emerging
Pantoea agglomerans	gram-negative	fastidious	No	emerging
Proteus mirabilis	gram-negative	non-fastidious	Yes	classical
Providencia stuartii	gram-negative	non-fastidious	Yes	classical
Pseudomonas aeruginosa	gram-negative	non-fastidious	Yes	classical
Serratia marcescens	gram-negative	non-fastidious	Yes	classical
Staphylococcus aureus	gram-positive	non-fastidious	Yes	classical
Streptococcus agalactiae	gram-positive	fastidious	No	classical
Ureaplasma urealyticum	NA - No cell wall	fastidious	No	emerging
**Coagulase-negative *Staphylococci* (CoNS) [*Staphylococcus epidermidis, Staphylococcus haemolyticus, Staphylococcus lugdunenesis, Staphylococcus saprophyticus*]**	gram-positive	non-fastidious	Yes	emerging
**Viridans group *Streptococci* (VGS) [*Streptococcus anginosus, Streptococcus oralis, Streptococcus pasteuranus*]**	gram-positive	fastidious	No	emerging

Microorganism	Bacterial Cell Wall (gram-negative[a] or gram-positive[a])	Growth (fastidious[b] or non-fastidious[b])	P-AST provides appropriate growth conditions for organism	Classification (classical[c] or emerging[c])
***Enterobacter* group [*Klebsiella aerogenes* (formally known as *Enterobacter aerogenes*), *Enterobacter cloacae*]**	gram-negative	non-fastidious	Yes	classical

References for the role of each organisms as pathogens available upon request

[a]gram-positive vs gram-negative: gram-positive = bacteria that give a positive result in the Gram stain test, which uses crystal violet dye to categorize organisms based on the thickness of the peptidoglycan layer of the cell wall; gram-negative = bacteria that give a negative result in the Gram stain test

[b]fastidious vs non-fastidious: fastidious = microorganism that has complex or particular environmental and/or nutritional requirements not met by standard urine culture/P-AST growth conditions; non-fastidious = microorganism with simple growth requirements met by standard urine culture conditions

[c]classical vs emerging: classical = pathogens traditionally associated with UTI; emerging = microorganisms being newly recognized as potential or confirmed uropathogens
